# A retrospective analysis of the incidence of severe adverse events among recipients of chiropractic spinal manipulative therapy

**DOI:** 10.1038/s41598-023-28520-4

**Published:** 2023-01-23

**Authors:** Eric Chun-Pu Chu, Robert J. Trager, Linda Yin-King Lee, Imran Khan Niazi

**Affiliations:** 1 New York Chiropractic and Physiotherapy Centre, EC Healthcare, 41/F Langham Place Office Tower, 8 Argyle Street, Kowloon, Hong Kong; 2 Connor Whole Health, University Hospitals Cleveland Medical Center, 11000 Euclid Ave, Cleveland, OH 44106 USA; 3 College of Chiropractic, Logan University, Chesterfield, MO 63017 USA; 4 School of Nursing and Health Studies, Hong Kong Metropolitan University, Kowloon, Hong Kong; 5 Centre for Chiropractic Research, New Zealand College of Chiropractic, Auckland, 1060 New Zealand

**Keywords:** Adverse effects, Rehabilitation

## Abstract

This study examined the incidence and severity of adverse events (AEs) of patients receiving chiropractic spinal manipulative therapy (SMT), with the hypothesis that < 1 per 100,000 SMT sessions results in a grade ≥ 3 (severe) AE. A secondary objective was to examine independent predictors of grade ≥ 3 AEs. We identified patients with SMT-related AEs from January 2017 through August 2022 across 30 chiropractic clinics in Hong Kong. AE data were extracted from a complaint log, including solicited patient surveys, complaints, and clinician reports, and corroborated by medical records. AEs were independently graded 1–5 based on severity (1-mild, 2-moderate, 3-severe, 4-life-threatening, 5-death). Among 960,140 SMT sessions for 54,846 patients, 39 AEs were identified, two were grade 3, both of which were rib fractures occurring in women age > 60 with osteoporosis, while none were grade ≥ 4, yielding an incidence of grade ≥ 3 AEs of 0.21 per 100,000 SMT sessions (95% CI 0.00, 0.56 per 100,000). There were no AEs related to stroke or cauda equina syndrome. The sample size was insufficient to identify predictors of grade ≥ 3 AEs using multiple logistic regression. In this study, severe SMT-related AEs were reassuringly very rare.

## Introduction

An adverse event (AE) is any unfavorable and unintended sign, symptom, or disease temporally associated with the use of medical treatment or procedure that may or may not be considered related to the medical treatment or procedure^1^. The current study focused on AEs related to spinal manipulative therapy (SMT) involving a thrust or impulse, a treatment commonly used by chiropractors to treat spinal conditions^2^. While mild SMT-related AEs such as transient soreness are better understood and reported to be common, there has been limited research examining the incidence of severe SMT-related AEs using a large sample supported by medical records data^3^.

Previous large studies have examined AEs occurring in relation to SMT from various professions, including chiropractic^4^, Chuna (traditional Korean manual therapy)^5^, and osteopathy^6^. Studies have estimated that severe AEs such as fractures, cauda equina syndrome, or cervical artery dissection occur between 1 per 2 million to 7 per 100,000 SMT treatments^3^. However, there have been several limitations in such studies, such as the use of a variety of customized definitions or grading systems for AEs, making this data challenging to interpret^4^. Some studies have relied on administrative claims data that lacks verification with the medical records^7^, report AEs caused by individuals unqualified to administer SMT^8,9^, or do not include patients’ baseline symptoms prior to the AE^10^. Finally, randomized controlled trials of SMT may underestimate the incidence of AEs as the exclusion criteria in these studies may omit at-risk patients^3^.

A further challenge in this research is a lack of understanding of which variables predict severe AEs related to SMT^3,11^. Previous research has identified predictors of mild or benign AEs, such as worker’s compensation or sick leave, higher level of disability, female sex, increasing age, and first SMT session^12–15^. However, it is not clear if these predictors apply to severe AEs. This is partly a limitation of sample size. Given that severe AEs are uncommon, none were analysed in the datasets of studies examining AE predictors^12–14,16^. Limited information on risk factors for severe AEs can be gleaned from case reports, which, for example, have described SMT-related fractures in patients with osteoporosis or unrecognized cancer^17^.

SMT is effective for neck and low back pain^18,19^ and is recommended by multiple clinical practice guidelines^20–22^. While many disciplines utilize SMT, such as physical therapists and osteopaths, chiropractors may be the predominant users of this treatment worldwide^23^. The most common reasons patients visit a chiropractor include spinal conditions, primarily low back and neck pain^24^.

While there are several methods of ascertaining AEs in relation to SMT, previous studies suggest that medical records alone are insufficient to capture AEs^25^. One possible reason is that patients may not report AEs directly to the clinicians due to not wanting to introduce tension into the doctor-patient relationship^25^. In contrast, other research has shown that patients are more likely to report AEs when asked open-ended questions about their personal experiences via various solicitation methods outside the clinical setting^26^. As such, a range of data sources, including patient-driven complaints, complaints solicited via questionnaires, and data from medical records may help better understand SMT-related AEs.

Given the limitations of previous research examining SMT-related AEs, this retrospective study aimed to investigate the incidence and types of adverse events occurring among patients receiving chiropractic SMT in integrated clinics in Hong Kong by searching a database that combined several reporting methods for AEs. Our primary hypothesis was that severe AEs would be rare and occur in less than 1 per 100,000 SMT treatments.

## Methods

### Study design

This study adhered to an a priori protocol registered in the Open Science Framework (https://osf.io/ub237)^27^. The Ethics Committee of the Chiropractic Doctors Association of Hong Kong (Causeway Bay, Hong Kong, IRB ID: CDA20220827) approved the study which included a waiver of patient consent. All methods were performed in accordance with the relevant guidelines and regulations. The current study was a retrospective database analysis of a complaints log including AEs, bolstered by clinical data obtained via chart review, from January 1, 2017, through August 31, 2022.

### Setting

Data originated from 30 affiliated chiropractic clinics with 38 chiropractors (New York Chiropractic & Physiotherapy Center, EC Healthcare, Hong Kong). These clinics are integrated into a larger healthcare organization, including several medical specialties and imaging and laboratory testing centers that utilize a shared medical records system.

### Data source

Data regarding AEs was obtained from a detailed complaint log that was routinely aggregated from several sources by a customer service department. One source of AEs in this log was a custom survey administered to patients after their 1st, 2nd, and 16th visits. This questionnaire was sent to patients or parents/guardians (in the case of minors) via a customized secure short message service (SMS) and included an open-ended question that asked patients to describe any “Other negative comments/side effects/service suggestions.” Questionnaires were administered in both Chinese and English. Smartphone use in Hong Kong is among the highest in the world, with almost 90% of individuals over age 10 using a smartphone^28^.

Additional AEs derived from follow-up phone calls by a personal health manager, which was made following the 1st, 2nd, and 16th visits. Personal health managers asked patients open-ended questions pertaining to their care (translated from Chinese): “How was your patient experience during the treatment?” and “Are you feeling any discomfort after the treatment?” If patients answered yes to the second question, the health manager asked further questions to characterize the discomfort: “Can you describe what you are feeling?” and “When did this start after treatment?” and “What were you doing when you had the symptoms?” and “How would you describe these symptoms, or any additional symptoms?” and “Where are the symptoms?” and “What is the intensity of the symptoms?” In addition, the health manager was trained to reassure patients regarding mild soreness following SMT and these symptoms were generally not pursued further as formal complaints or AEs. In contrast, any potential treatment-related symptoms greater than mild soreness were documented and forwarded to the customer service complaint log.

Clinical staff could also register an incident report that was aggregated within the customer service department log. The customer service department also routinely aggregated AEs reported from emails, non-solicited phone calls, customer service hotline and SMS, website contact information, social media and online forums, internet (e.g., Google) reviews, and others.

The customer service department grouped all clinical care-related complaints, including AEs, separately from non-clinical complaints (e.g., payment, wait time, miscommunications). In the case that customer service identified a clinical complaint, the chief operating officer, legal office, treating clinician, and patient personal health manager were notified such that they could adequately follow up with the patient (e.g., to determine if hospitalization or intervention was required). The complaints log included the complaint date, description, incident date, and source of complaint (e.g., SMS survey, email). Specific dates, names, and other patient identifiers were not extracted for the current study.

Data regarding AEs were corroborated by medical records data, which was identified by manually searching an electronic health records system (CSP, EC Healthcare, Hong Kong) to identify clinical information from notes, specialist visits, and/or diagnostic imaging reports. A patient identification number was used to cross-reference the medical records with the original complaint log.

Records queries and data extraction were performed by three information technology professionals who were blinded to the study hypotheses and extracted data into a pre-defined Microsoft Excel spreadsheet. These professionals entered data regarding the clinical complaints from the customer service log, which included a free-text description of the details of the AE as well as the duration of the AE and any follow-up care. Information technology professionals also transcribed de-identified data from each patient’s medical record including a free-text description of their chief complaint and history of present illness, examination findings, and treatment on the date of or immediately preceding the AE, as well as demographic data, pain severity, comorbidities, medications, primary diagnosis, SMT session AE occurred, and any available radiological impression related to the AE. Data were checked for accuracy by EC and later harmonized according to consistent terminology by RT which included conversion of comorbidities into the Chronic Conditions count, and translation of the SMT description into a standard nomenclature (i.e., region of spine, patient position, technique). AE grade was determined later.

### Participants

Patients of any age receiving SMT within any of the affiliated chiropractic clinics were included. Patients were required to receive SMT administered via manual thrust (i.e., a hands-on impulse applied to the spinal joints). Patients could also receive other therapies in addition to SMT. However, mechanical spinal traction, joint mobilization, and soft tissue therapies were not considered chiropractic SMT as part of this study. Any chief complaint was included to maximize the sample size (e.g., neck pain, low back pain).

This study defined an AE as any new complaint which was not present at baseline or a worsening of a presenting complaint. These criteria are similar but less restrictive than previous definitions for AEs, which required a minimum percentage increase in symptoms (i.e., > 30%)^14,29–31^. The authors considered that the previous quantitative definition may be difficult to apply to non-painful or subjective symptoms, therefore a more general definition was used. For example, dizziness following SMT in a patient who presented to the chiropractor with only localized neck pain would be considered a new complaint and categorized as an AE. Examples of worsening of a complaint that would be considered an AE would be a greater pain severity, additional radiation of symptoms, or lower extremity weakness or numbness following SMT in a patient who presented with localized low back pain.

As providers in the study clinics often utilize multimodal treatments, our approach included AEs which occurred among patients receiving SMT in isolation as well as AEs that occurred among patients receiving SMT alongside other therapies on the same date of service. However, AEs that occurred in relation to a non-SMT therapy on a treatment day that SMT was not provided were not considered SMT-related. A similar conservative approach to including AEs among patients receiving contemporaneous multimodal care was also utilized in a similar study^5^. For example, if a patient received thoracic SMT in addition to a back massage provided by a mechanical device during the same date of care, then developed a vertebral fracture, this was considered a potential SMT-related AE. In such a hypothetical example, it would not be feasible to discern which treatment (i.e., SMT or massage) was more likely to be responsible for the fracture.

While various schemes have been used previously to grade the severity of AEs in relation to SMT^4^, the current study utilized the Common Terminology Criteria for Adverse Events (CTCAE). Previous authors have utilized this system to grade the severity of AEs related to SMT^5,32–34^. CTCAE is becoming a standard measurement system and is used in other fields, such as cancer therapies^35^, pharmacology^36^, and surgery^37^.

### Variables

#### AE grade

As a primary outcome, this variable was recorded using the CTCAE rubric (Table 1) ^1^. Co-authors RT and LL reviewed the available AE data for each patient and independently determined a grade for each. Each author based their AE grade on data available in the de-identified extraction sheet, which included all extracted variables in the present study. While all data were considered within AE grading, data regarding follow-up care, which described any hospitalization or necessary intervention in relation to the AE, was particularly relevant. In addition, both authors were provided with the CTCAE v5.0 document which provides detailed examples of application of this rubric to specific types of AEs^1^. Prior to grading, the published example for spinal fracture was shared among investigators, which describes a grade 3 AE for spinal fracture as involving “severe back pain; hospitalization or intervention indicated for pain control (e.g., vertebroplasty); limiting self care ADL; disability.”

**Table 1:** Common terminology criteria for adverse events. Table adapted from the US Department of Health and Human Services ^1^.

Grade	Description
Grade 1	**Mild**; asymptomatic or mild symptoms; clinical or diagnostic observations only; intervention not indicated
Grade 2	**Moderate**; minimal, local or noninvasive intervention indicated; limiting age-appropriate instrumental ADL*
Grade 3	**Severe** or medically significant but not immediately life-threatening; hospitalization or prolongation of hospitalization indicated; disabling; limiting self-care ADL**
Grade 4	**Life-threatening** consequences; urgent intervention indicated
Grade 5	**Death** related to AE

*Instrumental ADL refers to preparing meals, shopping for groceries or clothes, using the telephone, managing money, etc.

**Self-care ADL refers to bathing, dressing and undressing, feeding self, using the toilet, taking medications, and not bedridden.

Abbreviations: activities of daily living (ADL).

#### Patient characteristics

Patient demographics, including age at the time of presentation and sex, were reported. In addition, comorbidities appearing in the Centers for Medicare and Medicaid definition for Chronic Conditions (e.g., osteoporosis, cancer, diabetes, stroke) were recorded^38^. Patients’ medications, chief complaint, symptoms preceding the AE, and initial pain severity on the numeric pain rating scale (from 0 to 10; 10 is the most severe pain), were recorded in free text format.

#### AE characteristics

The visit of SMT in which the AE occurred was reported. For example, an AE occurring at the 1st visit would be recorded as a 1, the 2nd visit would be a 2, and so on. Details regarding the AE were described in a free text narrative, including the patient’s symptoms, details regarding imaging and or medical care, and any follow-up care. The origin of the AE was recorded depending on its source, e.g., a patient survey or email. The duration of the patients’ symptoms was recorded as days (i.e., 1–6 days), weeks (≥ 7 days), months (≥ 1 month), or ongoing/permanent.

#### Missing data

If the above data items were unclear or unavailable from the complaint log or medical record, this was noted in the extraction sheet. However, almost all data items were available. The exact type of medication they took was unclear for one patient with a neurodegenerative disease.

### Statistical analysis

A sample size of 7,299 for our primary objective was calculated using data from a similar study that identified 50 AEs per 2,682,258 manipulations, yielding an incidence of 0.000019 for AEs^5^. The formula n = [Z2P(1 − P)]/(d2) was utilized wherein Z equals the Z statistic for the level of confidence of 95% (i.e., 1.96), P is the probability of 0.000019, and d is the required precision, of 0.0001 or 0.01%^39^. Descriptive statistics were conducted using Microsoft Excel (Version 2211) while 95% confidence intervals for AE incidence were calculated using the Wilson Score interval^40^ via a free web tool designed for this purpose^41^. Incidence of AEs per grade was calculated by dividing the raw number of AEs in each grade by the total number of SMT sessions. Incidence per 100,000 treatments was calculated by multiplying this value by a factor of 100,000.

For our secondary objective, a sample size of at least 40 total AEs (i.e., ≥ 40), with at least one AE of grade 3 or higher was required for multiple logistic regression to determine independent predictors of grade 3 or higher AE, estimating a minimum 10 cases per variable (i.e., 10*4 = 40)^42^. Considering a potentially limited sample size, a sparing regression model with only four key covariates were selected, including age, sex, number of comorbidities, and SMT visit.

### Ethical approval and informed consent

The Ethics Committee of the Chiropractic Doctors Association of Hong Kong (Causeway Bay, Hong Kong, IRB ID: CDA20220827) approved the study and waived the need for informed consent as de-identified data were used.

## Results

### AE grading

The authors RT and LL had 85% agreement of independent AE grades, with six of 39 cases having discrepant scores. All discrepant scores were either graded “1” or “2” and ultimately were resolved via mutual discussion.

### Participants

**Figure 1:**
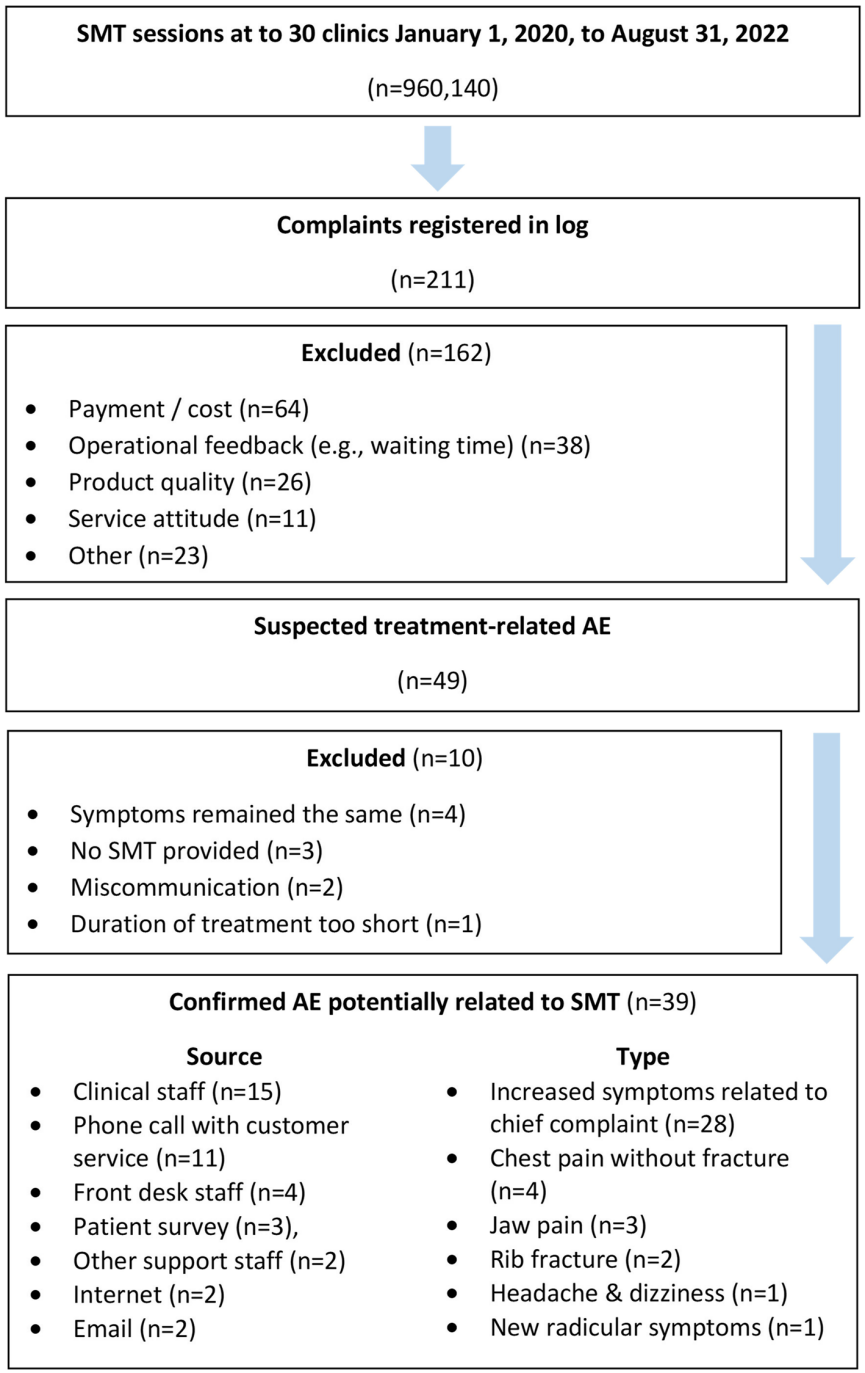
Identification of patients with adverse events related to spinal manipulative therapy. Abbreviations: adverse event (AE), spinal manipulative therapy (SMT).

During the study period there were 960,140 treatment sessions involving manual thrust chiropractic SMT across 54,846 unique patients (Fig. 1). During this time, there were 211 complaints registered in the customer service log. Of these complaints, 49 were related to clinical care and thus were screened according to our selection criteria for potential SMT-related AEs. Ten suspected AEs were excluded, as the patient’s symptoms remained the same (n = 4), a physiotherapeutic modality was provided rather than SMT (n = 3), there was a patient miscommunication regarding diagnosis or treatment rather than an AE (n = 2), or a patient complained that the treatment provided was too short (n = 1). Thirty-nine patients had a confirmed AE, which was potentially related to SMT. Among the study population, 13.8% of patients (n = 7,569) had registered a response to at least one SMS questionnaire while 95.5% (n = 52,377) had registered a response to at least one follow-up phone call with the personal health manager.

The mean patient age was 50.8 ± 18.3 (s.d.), with 74% of patients being female. The most common chief complaint was low back pain (46%), followed by spine pain occurring in multiple regions such as the neck and low back (28%), neck pain (18%), thoracic spine pain (3%), shoulder pain (3%), and hip pain (3%). A minority of patients had a comorbidity listed as a chronic condition per Medicare/Medicaid (36%), while only 13% had more than one chronic condition. Eighteen patients (46%) were taking at least one medication. Mean initial pain severity was 5.9 ± 2.0 (s.d.).

### AE details

The 39 AEs potentially related to chiropractic SMT included increased symptoms related to the patient’s chief complaint (n = 28), chest pain without a fracture on imaging (n = 4), jaw pain (n = 3), rib fracture confirmed by imaging (n = 2), headache and dizziness without evidence of stroke (n = 1), and new radicular symptoms (n = 1). Of the 39 AEs, grade 2 were most common (n = 32, 82%), followed by grade 1 (n = 5, 13%), and grade 3 (n = 2, 5%). There were no cases of stroke, transient ischemic attack, vertebral or carotid artery dissection, cauda equina syndrome, or spinal fracture.

The incidence of AEs (95% CI) was 0.52/100,000 (0.00, 1.13) for grade 1, 3.33/100,000 (2.22, 4.45) for grade 2, and 0.21/100,000 (0.00, 0.56) for grade 3 AEs. AEs occurred at a mean of 8.9 ± 8.6 visits (s.d.). Only 18% of AEs occurred after the 1st SMT session. AEs most often lasted days (74%), followed by hours (21%), weeks (3%), and months (3%). No AEs were reported to be permanent.

AEs were most often obtained from reports from the clinical staff (38%), followed by a phone call with customer service (28%), the front desk staff at the clinic (10%), SMS patient survey (8%), other support staff (5%), internet (5%), and email (5%).

### SMT description

The most common type of SMT implicated in AEs was supine cervical manipulation, occurring in 39% of cases, followed by lumbar and sacroiliac manipulations performed prone using a drop table (i.e.., involving a release mechanism whereby the table moves to a lower position during SMT; 13%), thoracic manipulations performed with the patient prone (18%), lumbar manipulations performed prone using a drop table (5%), thoracic manipulations performed supine (3%), and lumbar side posture manipulations (3%). Fourteen patients (36%) also received another type of spinal manual therapy or traction. As a percentage of total patients (n = 39), these included lumbar flexion-distraction (15%), massage (10%), and mechanical traction (10%).

### Risk factors

Although there was an insufficient sample to robustly examine independent predictors of severe AEs, possible contributing factors were identified among the reported AEs. Both cases of rib fracture occurred in females over 60 with a history of osteoporosis, following SMT performed to the thoracic spine with the patient prone. Among the four cases of chest pain without fracture evident on radiography, two patients also had a history of osteoporosis.

Among the three AEs involving jaw pain, two patients had a history of dental procedures that were potentially relevant. In one grade 2 AE, the patient had a known history of degenerative cervical spinal stenosis identified via magnetic resonance imaging, which may have accounted for symptom exacerbation after SMT. A potential risk factor noted in one AE was that the patient reported a prior AE after receiving cervical traction at another facility.

In three cases involving multimodal treatments, patients believed their symptoms began after a specific therapy other than SMT (n = 3, 8%). These cases were either grade 1 (n = 2) or grade 2 (n = 1) AEs. However, the exact cause of symptoms was not possible to verify as patients were receiving a combination of treatments on the same day. In two cases, patient-reported symptoms began after mechanical spinal traction (one case was cervical traction, the other was lumbar). In another case, symptoms began following massage therapy to the neck muscles.

### Regression model

As the sample size was below the minimum threshold (i.e., 39 instead of 40 AEs), a logistic regression model was not conducted to identify independent predictors of grade 3 or greater AEs.

## Discussion

This retrospective database analysis of AEs potentially related to chiropractic SMT at integrated clinics in Hong Kong included a large sample size with detailed patient information corroborated by medical records data and was based on an a priori protocol created by a multidisciplinary research team. The incidence of grade 3 (severe) or greater AEs was less than 1 per 100,000 SMT sessions, which supported our study hypothesis. To our knowledge, this study represents one of the largest to examine potential SMT-related AEs.

The estimated incidence of severe AEs potentially related to SMT are consistent with a previous review which estimated severe AEs occurred between 1 per 2 million and 7 per 100,000 SMT treatments^3^. However, this previous data was based on large cohort, case–control, or case-crossover studies^3^. In comparison, many chart review or survey studies examining AEs related to chiropractic SMT have had a smaller sample size (i.e., n ≤ 50,276 SMT treatments) and did not identify any severe AEs^12–15,30,31,43^. One recent large study, which examined the incidence of AEs related to Chuna manipulation, reported an incidence of severe AEs of 0.04 (95% CI 0.00, 0.16) per 100,000 treatment sessions in a sample of 2,682,258 manipulations^5^.

Rib fractures were the only severe AEs in the current study. According to a previous study, chiropractic SMT applied to the thoracic spine with the patient prone (which was the method used in the current study in cases of severe AEs) produces a reaction force against the chest from the chiropractic table, causing up to 4.5% compression of the chest anterior to posterior depth^44^. While such forces are estimated to be safe in healthy subjects^44^, such testing has not been conducted in individuals with reduced bone density^45^. A previous case series suggested that low bone mineral density is a risk factor for rib fracture after chiropractic SMT^45^. A rib fracture is also a recognized cause for malpractice litigation claims against chiropractors and, in one study, accounted for two of 48 claims in the United States (i.e., 4.2%)^46^. In the current study, the incidence of rib fractures was comparable to that identified in a previous study examining Chuna manipulation (i.e., 0.41; 95% CI 0.21, 0.70)^5^. However, in the current study, both cases of rib fracture were recorded as grade 3 (severe) AEs, whereas in the previous study, they were recorded as grade 2. This difference is valid, given that rib fractures have varying degrees of severity^47^.

This study highlights that chiropractors should be aware of the risk factors for low-trauma rib fracture in older patients, which include osteoporosis, older age, previous fall, and previous rib fracture^48^, and other risk factors for possible low bone mineral density, such as a sedentary lifestyle, alcohol intake or smoking status, or prolonged use of glucocorticoids^45^. For every 0.15 g/cm^2^ loss of femoral neck bone mineral density, there is an approximately twofold increase in the risk of low trauma rib fracture in both men and women aged 60 and older^48^. In a retrospective cohort study using administrative claims and including over one million patients age 66 and older in the United States, receiving chiropractic SMT increased the likelihood of fracture in patients with osteoporosis (odds ratio 1.66; 95% CI 1.16, 2.37)^49^. Accordingly, chiropractors should be cautious when treating older patients or those with low bone mineral density or its risk factors. As a general rule, forceful SMT is contraindicated in these patients^50^.

Aside from reduced bone density, underlying bone pathology presents a risk factor for severe AEs following SMT. A previous systematic review reported three cases of AE following SMT in patients who had previously undiagnosed spinal metastasis^17^. Two patients developed vertebral fracture, while the other developed paralysis from the waist down^17^. While no AEs related to undiagnosed spinal metastasis were identified in the current study, chiropractors should nonetheless remain vigilant to identify patients with bone pathology such as metastasis to circumvent SMT-related AEs.

Further research on this topic should be performed. A prospective multicenter registry of patients receiving chiropractic SMT would allow an estimate of the incidence and independent predictors of AEs while maximizing the available sample size. Examples of practice-based research networks, including chiropractic providers, include BraveNet in the United States^51^, the Australian Chiropractic Research Network^52^, and the Swiss chiropractic practice-based research network^53^. In addition, the current study could be updated in another three to four years which may allow sufficient sample size to identify independent predictors of severe AEs.

### Strengths and limitations

This study was based on an a priori protocol designed by a multidisciplinary author team to reduce bias. While there was a large sample of over 960,000 SMT treatments provided during the study window, the sample size of AEs was insufficient to identify independent predictors of grade 3 or higher AEs.

Although the current study's open-ended questionnaire and phone call methods are based on recommended methods to ascertain AEs^26^, the strategies have not been formally validated. Although the response rate to the follow-up phone calls was high, the response to the SMS questionnaire was low. It is possible that severe AEs occurred but were not reported or recorded through these or other methods of ascertainment (e.g., via clinician report, legal complaint, email).Recorded AEs were each corroborated by imaging reports and other clinical data, thus representing true positives. However, the rate of false negatives is unknown (e.g., AEs that were missed by our database analysis).

The current study methodology focused on ascertaining AEs of at least a severe grade and was not optimally designed to ascertain mild AEs. Mild AEs are known to be common following SMT, occurring after 23% to 83% of treatments^3^. Accordingly, the incidence of mild AEs in the present study is much too low to be considered accurate. In the present study, the chiropractors informed patients that mild AEs were common and transient prior to administering SMT per clinical practice standards. In addition, patients signed an informed consent document which similarly described that they may experience “increase of pain” following SMT. Finally, when patients reported mild soreness after SMT to personal health managers during follow-up phone calls, the health managers first reassured patients that these symptoms were typical and generally did not register a formal complaint or AE. Between the chiropractor’s verbal and written informed consent and reassurance from the personal health manager, we suspect that patients were much less inclined to formally report a mild AE (compared to a severe AE). However, we still reported the incidence of mild AEs for transparency and completeness.

While several data items were included, additional variables may have been helpful. Disability levels, as ascertained from patient-reported outcome assessments, were not utilized in the current study as a variety of measures (e.g., Oswestry disability index, World Health Organization Quality of Life score) were used over the study time window. Further, practitioner-related variables such as years’ experience, time spent with patients, or additional certifications or diplomate status were not considered. A prospective cohort study design including these data items would be a superior study design for examining the incidence and severity of AEs.

Our sampling method did not include detailed data regarding the source population without AEs (e.g., the 54,846 patients receiving SMT). Further, a detailed patient-level raw dataset could not be presented. These datasets were not permitted by our protocol and ethics approval, and individual patient data was not feasible to show as this could have enabled individual patients to be identified.

The chiropractic SMT examined in the current study may be generalizable to other chiropractic practices as the most common chief complaints included spinal pain, which is the most common condition treated by chiropractors worldwide^24^. However, there may be differences in the patient populations, comorbidity prevalence, and healthcare systems between Hong Kong and elsewhere. Further, results may not be comparable to other practitioner-delivered types of SMT, such as those administered by osteopaths or physiotherapists.

The current study did not account for the magnitude of force delivered during chiropractic SMT, which would be difficult to ascertain from the clinical record. While chiropractic clinicians often account for patients’ comorbidities when administering SMT, for example, using less force for patients with osteoporosis^54^, such precaution or lack thereof could have been a critical unmeasured variable.

Patients who developed rib fractures or chest pain after SMT either underwent radiography and/or computed tomography. It is possible that those receiving only radiography had a false negative imaging study^55^. However, other diagnoses, such as rib contusion, which may be identified via magnetic resonance imaging^56^, or costochondritis, are also possible explanations.

## Conclusions

This current study, which retrospectively studied a large dataset from integrated chiropractic clinics in Hong Kong, found that severe AEs potentially occurring in relation to SMT were rare, yielding an incidence of 0.21 per 100,000 SMT sessions. No AEs were identified that were life-threatening or resulted in death. The sample size of 39 AEs across 960,140 SMT sessions in 54,846 patients was insufficient to identify independent predictors of severe AEs. Further research on this topic is needed, possibly via a practice-based research network which could increase the sample size and allow for such analysis.

## Data Availability

The datasets generated during and/or analysed during the current study are not publicly available due to ethical restrictions and for de-identification purposes but are available from the corresponding author on reasonable request.
